# Climate Controls on the Spatial Variability of Vegetation Greenup Rate across Ecosystems in Northern Hemisphere

**DOI:** 10.3390/plants11212971

**Published:** 2022-11-03

**Authors:** Zhoutao Zheng

**Affiliations:** Lhasa Plateau Ecosystem Research Station, Key Laboratory of Ecosystem Network Observation and Modeling, Institute of Geographic Sciences and Natural Resources Research, Chinese Academy of Sciences, Beijing 100101, China; zhengzt@igsnrr.ac.cn

**Keywords:** greenup rate, eddy covariance, phenology, vegetation dynamics, spatial variability, gross primary productivity

## Abstract

Variations in individual phenological events in response to global change have received considerable attentions. However, the development of phenological stages is relatively neglected, especially based on in situ observation data. In this study, the rate of vegetation greenup (Vgreenup) across the Northern Hemisphere was examined for different plant functional types (PFTs) by using eddy covariance flux data from 40 sites (417 site-years). Then, the controls of climatic variables on the spatial distribution of Vgreenup across PFTs were further investigated. The mean Vgreenup was 0.22 ± 0.11 g C m^−2^ day^−2^ across all sites, with the largest and lowest values observed in cropland and evergreen needle-leaf forest, respectively. A strong latitude dependence by Vgreenup was observed in both Europe and North America. The spatial variations of Vgreenup were jointly regulated by the duration of greenup (Dgreenup) and the amplitude of greenup (Agreenup). However, the predominant factor was Dgreenup in Europe, which changed to Agreenup in North America. Spring climatic factors exerted significant influences on the spatial distribution of Vgreenup across PFTs. Specifically, increasing temperature tended to shorten Dgreenup and promote Agreenup simultaneously, resulting in an acceleration of Vgreenup. Dryness had a depression effect on Vgreenup for the whole study area, as exhibited by a lower Vgreenup with increasing vapor pressure deficit or decreasing soil moisture. However, Vgreenup in North America was only significantly and positively correlated with temperature. Without the limitation of other climatic factors, the temperature sensitivity of Vgreenup was higher in North America (0.021 g C m^−2^ day^−2^ °C^−1^) than in Europe (0.015 g C m^−2^ day^−2^ °C^−1^). This study provides new cognitions for Vgreenup dynamics from in situ observations in complement to satellite observations, which can improve our understanding of terrestrial carbon cycles.

## 1. Introduction

Vegetation phenology is the study of the timing of recurring plant life cycle events that are driven by environmental factors [1,2,3]. It not only is a key indicator of climate change but also exerts important influence on terrestrial ecosystem by altering carbon, nitrogen, and water cycles [4,5]. Many studies have addressed the relationship between the timing of individual phenological events and their drivers [6,7,8]. Under global warming, both remote sensing and ground-based observations reveal the substantial advancement of greenup, delay of leaf senescence, and extension of the growing season [9,10,11,12,13]. However, compared with individual phenological event, the development of the phenological stages has received inadequate attention.

The rate of greenup (Vgreenup) was proposed to facilitate the understanding and description of vegetation greenup development [14,15], which is calculated as the ratio of the duration of greenup (Dgreenup) to the amplitude of greenup (Agreenup). The variation in Vgreenup has strong linkage with vegetation growth and carbon sequestration by altering vegetation photosynthesis, respiration, and even autumn leaf senescence [16]. Vgreenup also influences vegetation cover and can further provide feedback to the climate system by regulating the exchange of water and energy between the land surface and atmosphere [17,18]. Therefore, the dynamics of greenup development and the underlying mechanism need to be thoroughly investigated to improve regional and global carbon cycles modeling performances.

Increasing Vgreenup has been reported for the Northern Hemisphere and the globe based on remote sensing observations [19,20,21]. Using the leaf area index (LAI) dataset, Wang et al. [19] found that the global average Vgreenup had increased by 0.003 m^2^ m^−2^ day^−1^ in response to climate change and human land management from 1982–2015, whereas the average Dgreenup had shortened by 1.4 days, and Agreenup had increased by 0.116 m^2^ m^−2^. Another study also showed an increasing trend of Vgreenup induced by the warming at high latitudes above 60° N, from 1982–2016, based on LAI data [20]. Despite the globally accelerated Vgreenup, some areas experienced decreasing Vgreenup. For example, Vgreenup declined in Northeast China between 1982 and 2015 due to the impact of frost [22]. Therefore, Vgreenup will not exhibit identical trends under distinct environments, so it is crucial to reveal the underlying mechanism. Though the relationships between Vgreenup and some climatic factors (such as temperature, precipitation, and radiation) have been revealed, more comprehensive research including additional climatic factors is still needed to better understand the driving mechanism underlying Vgreenup. For example, dryness and wind speed were confirmed to have significant effects on vegetation phenology and productivity [23,24].

Although remote sensing data have the advantages of extensive spatial coverage and high temporal frequencies, their results can be highly biased by data quality, due to snow and cloud contamination, sensor degradation bias, and the mixed pixel problem, especially in sparse vegetation areas [2]. Moreover, high limitations exist for revealing the driving mechanism based on remote sensing techniques without ground validation. Continuous ground observation can provide data for a deepened understanding of the driving forces and mechanism underlying greenup development. However, research on Vgreenup based on ground observation is still lacking. At present, the acquisition of ground-observed phenology data mainly originates from manual measurements, repeated digital photography, and eddy covariance measurements [25]. Among these methods, eddy covariance measurements at a flux tower form a reliable and widely used way to extract phenology at a landscape scale [26].

This study aimed to investigate the spatial variations in Vgreenup and its driving climatic factors by using eddy covariance data from 40 flux tower sites (417 site-years) in the northern temperate and boreal regions (>30° N). The specific objectives were: (1) to compare Vgreenup for different plant functional types (PFTs); (2) to elucidate the spatial pattern of Vgreenup and its underlying mechanism; (3) to reveal the difference in Vgreenup variation and driving factors between continents (Europe and North America).

## 2. Results

### 2.1. Measures of Vgreenup among PFTs

**Figure 1:**
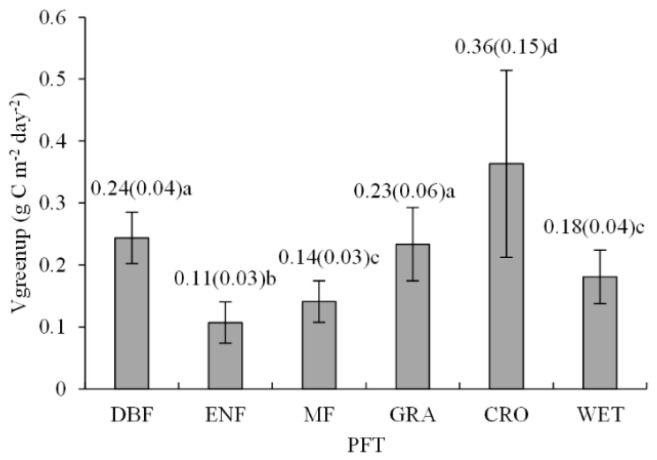
Vgreenup in each plant function type (PTF). The number above each PTF is the mean value of Vgreenup, and the number in the parentheses is the standard deviation of Vgreenup for each PTF. Different letters indicate significant difference (*p* < 0.05) in one-way ANOVA using LSD multiple comparisons among PTFs. DBF, deciduous broadleaf forest; ENF, evergreen needle-leaf forest; MF, mixed forest; GRA, grassland; CRO, cropland; WET, wetland.

Vgreenup ranged from 0.06 to 0.54 g C m^−2^ day^−2^ across the 40 eddy covariance sites (Table 1), with a mean of 0.22 ± 0.11 g C m^−2^ day^−2^. The difference in Vgreenup was also significant among PFTs (*F* = 123.64, *p* < 0.001) (Figure 1). Cropland (CRO) exhibited the largest Vgreenup, followed by deciduous broadleaf forest (DBF), grassland (GRA), wetland (WET), mixed forest (MF), and evergreen needle-leaf forest (ENF) (Figure 1). Besides, Vgreenup varied more in CRO than in the other PFTs, as shown by a greater standard deviation. Vgreenup in GRA was comparable to that in DBF. In addition, there was no significant difference in Vgreenup between MF and WET.

### 2.2. Spatial Distribution of Vgreenup

**Figure 2:**
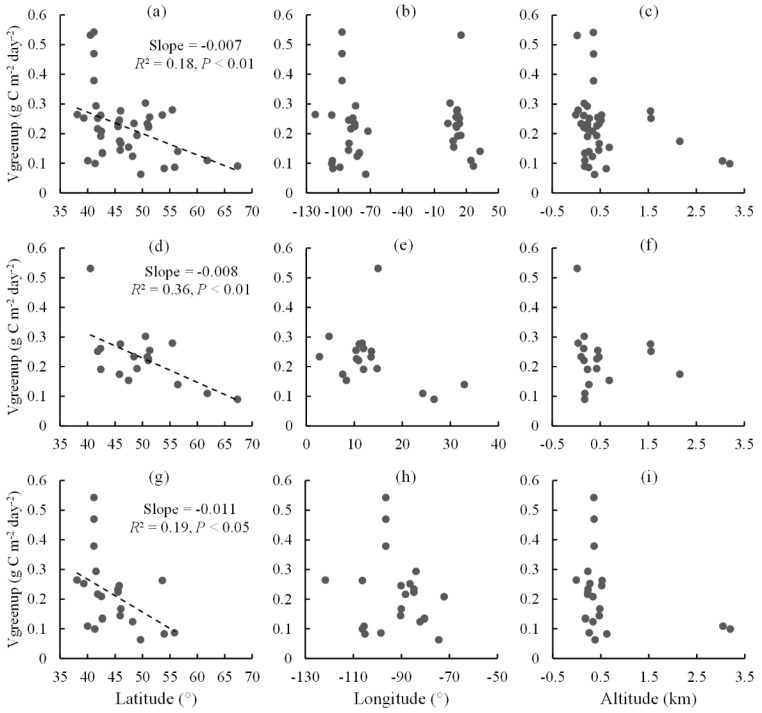
The relationships between Vgreenup and geographical factors. (**a**–**c**) The geographical patterns of Vgreenup across all sites in Europe and North America; (**d**–**f**) the geographical patterns of Vgreenup across all sites in Europe; (**g**–**i**) the geographical patterns of Vgreenup across all sites in North America.

Vgreenup had a clear spatial pattern, which showed a strong dependence on latitude across all sites but no dependence on longitude and altitude (Figure 2a–c). Vgreenup decreased 0.007 g C m^−2^ day^−2^ with an increase in latitude per degree, which was different from the increasing Vgreenup trend with latitude observed by remote sensing in a previous study [21]. Besides, the strong dependence of Vgreenup on latitude was also observed in North America as well as in Europe (Figure 2d,g). Vgreenup decreased 0.008 and 0.011 g C m^−2^ day^−2^ per degree latitude increase in Europe and North America, respectively. There was no evident dependence of Vgreenup on longitude and altitude on the two continents separately.

### 2.3. Spatial Relationships between Vgreenup and Its Components

**Figure 3:**
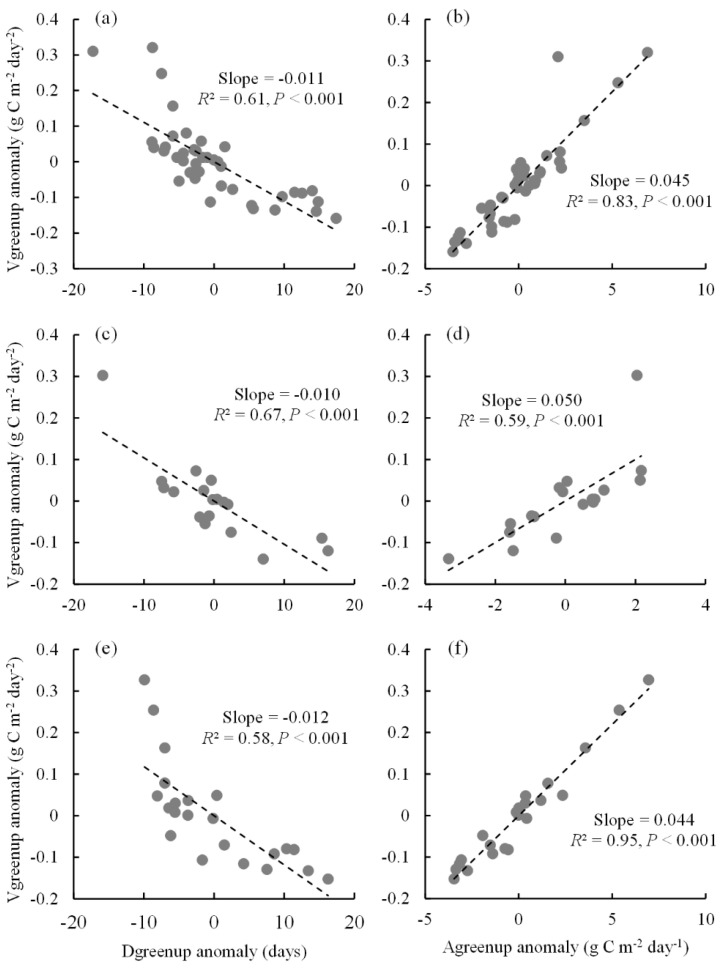
The spatial relationships between Vgreenup anomalies and Dgreenup anomalies as well as Agreenup anomalies. (**a**,**b**) The spatial relationships across all sites in both Europe and North America; (**c**,**d**) the spatial relationships across all sites in Europe; (**e**,**f**) the spatial relationships across all sites in North America.

Vgreenup anomalies showed a significantly negative correlation with Dgreenup anomalies and a significantly positive correlation with Agreenup anomalies across all sites in the whole study area (Figure 3a,b). This was different from the findings of a previous satellite-based study [19], in which Vgreenup was positively correlated with both Dgreenup and Agreenup at a global scale. Across all sites in this study, the linear spatial correlation between Vgreenup anomalies and Dgreenup anomalies (*R*^2^ = 0.61, *p* < 0.001) was relatively weaker than that between Vgreenup anomalies and Agreenup anomalies (*R*^2^ = 0.83, *p* < 0.001), which implies the predominant role of Agreenup in the whole study area. The relative contributions of Dgreenup and Agreenup to the spatial variation of Vgreenup were different between Europe and North America (Figure 3c–f). Dgreenup anomalies exhibited a higher correlation (*R*^2^ = 0.67, *p* < 0.001) in explaining the spatial variation of Vgreenup anomalies in comparison to Agreenup anomalies (*R*^2^ = 0.59, *p* < 0.001) in Europe (Figure 3c,d). However, Vgreenup anomalies correlated more strongly with Agreenup anomalies (*R*^2^ = 0.95, *p* < 0.001) than with Dgreenup anomalies (*R*^2^ = 0.58, *p* < 0.001) in North America (Figure 3e,f), which indicated that the spatial variation of Vgreenup was more predominated by Agreenup.

### 2.4. Spatial Relationships between Vgreenup and Climatic Factors

**Figure 4:**
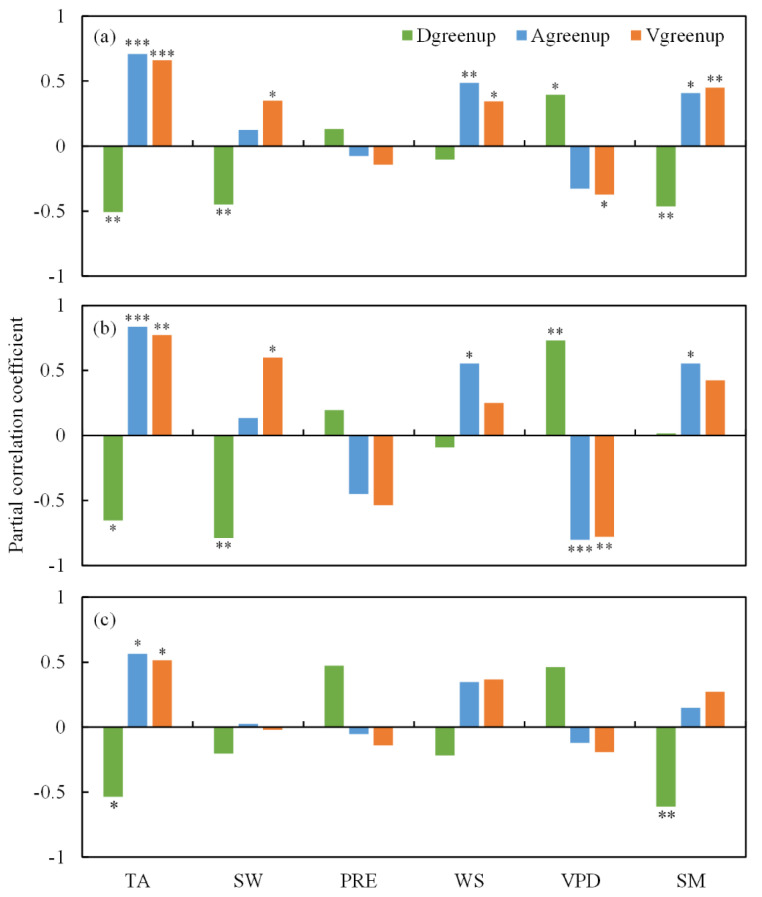
The partial correlation coefficients between spring climatic factors and Dgreenup, Agreenup, and Vgreenup. (**a**) The partial correlation coefficients across all sites in both Europe and North America; (**b**) the partial correlation coefficients across all sites in Europe; (**c**) the partial correlation coefficients across all sites in North America. TA, SW, PRE, WS, VPD, and SM represent temperature, shortwave radiation, precipitation, wind speed, vapor pressure deficit, and soil moisture, respectively. *, ** and *** indicate significant correlation at *p* < 0.05, 0.01 and 0.001 levels, respectively.

The spatial pattern of Vgreenup was regulated by multiple spring climatic factors across all sites in the whole study area (Figure 4a). Temperature (TA) played a predominant role in controlling the spatial variation in Vgreenup across all sites, with a significantly positive partial correlation (*R* = 0.66, *p* < 0.001), followed by soil moisture (SM) (*R* = 0.45, *p* < 0.01), vapor pressure deficit (VPD) (*R* = −0.37, *p* < 0.05), shortwave radiation (SW) (*R* = 0.35, *p* < 0.05), and wind speed (WS) (*R* = 0.35, *p* < 0.05). However, no significant relationship was found between precipitation (PRE) and Vgreenup. TA was significantly negatively correlated with Dgreenup (*R* = −0.51, *p* < 0.01) and was positively correlated with Agreenup (*R* = 0.71, *p* < 0.001), which indicated that increasing TA would shorten Dgreenup and promote Agreenup. Meanwhile, SM and VPD showed contrasting effects on Dgreenup as well as on Agreenup. SM exhibited a negative correlation with Dgreenup (*R* = −0.46, *p* < 0.01) and a positive correlation with Agreenup (*R* = 0.41, *p* < 0.05), while VPD was positively correlated with Dgreenup (*R* = 0.41, *p* < 0.05) and negatively correlated with Agreenup (*R* = −0.33, *p* > 0.05). This indicated that increasing VPD and decreasing SM would lengthen Dgreenup and lower Agreenup. Besides, a negative effect of SW on Dgreenup (*R* = −0.45, *p* < 0.01) and a positive effect of WS on Agreenup were also observed (*R* = 0.49, *p* < 0.01). It could also be observed that PRE had a minor influence on Dgreenup and Vgreenup.

In Europe, the effects of spring climatic factors on Vgreenup and its components were similar to those in the whole study area. Except for PRE, all spring climatic factors showed significant correlations with either Dgreenup or Agreenup in Europe (Figure 4b). A significantly positive effect of TA (*R* = 0.77, *p* < 0.01) and SW (*R* = 0.60, *p* < 0.05) on Vgreenup and a significantly negative effect of VPD on Vgreenup (*R* = −0.78, *p* < 0.01) could be found. However, no significant relationship was found between Vgreenup and spring climatic factors except for TA (*R* = 0.51, *p* < 0.05) in North America (Figure 4c). TA also showed significant relationships with Dgreenup (*R* = −0.54, *p* < 0.05) and Agreenup (*R* = 0.56, *p* < 0.05) in North America. Besides, SM was significantly and negatively correlated with Dgreenup (*R* = −0.61, *p* < 0.01) but exerted no significant influence on Agreenup or Vgreenup in North America (Figure 4c).

**Figure 5:**
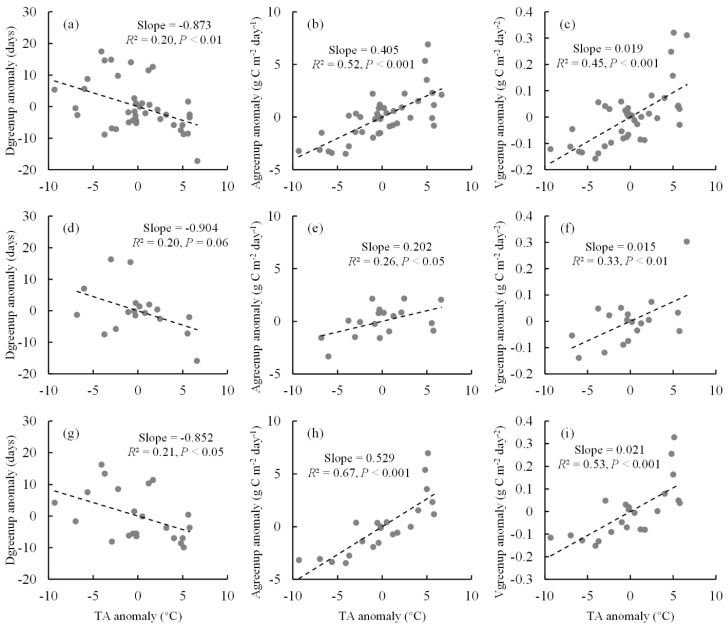
The spatial relationships between temperature anomalies and Dgreenup anomalies, Agreenup anomalies, and Vgreenup anomalies. (**a**–**c**) The spatial relationships across all sites in both Europe and North America; (**d**–**f**) the spatial relationships across all sites in Europe; (**g**–**i**) the spatial relationships across all sites in North America.

This study also analyzed the temperature sensitivity of Vgreenup and its components (Figure 5). In response to a 1 °C increase in TA, Dgreenup would shorten by 0.87 days across all sites of the whole study area, while Agreenup and Vgreenup would increase by 0.41 g C m^−2^ day^−1^ and 0.019 g C m^−2^ day^−2^, respectively (Figure 5a–c). The temperature sensitivity amplitudes in relation to Vgreenup and its components were different between Europe and North America (Figure 5d–i). The temperature sensitivity of Dgreenup was slightly greater in Europe (−0.90 days °C^−1^) than in North America (−0.85 days °C^−1^) (Figure 5d,g). However, the effect of TA on Agreenup was much stronger in North America than in Europe. With a 1 °C increase in TA, Agreenup would increase by 0.20 and 0.53 g C m^−2^ day^−1^ in Europe and North America, respectively (Figure 5e,h). Therefore, a greater temperature sensitivity of Vgreenup was observed in North America (0.021 g C m^−2^ day^−2^ °C^−1^) than in Europe (0.015 g C m^−2^ day^−2^ °C^−1^) (Figure 5f,i).

## 3. Discussion

Many previous studies have reported the distribution patterns along latitude, longitude, and altitude for vegetation phenology and productivity in adaptation to climate changes [27,28,29]. This study revealed the spatial pattern of Vgreenup across PFTs in the northern temperate and boreal regions. A strong dependence on latitude was observed for Vgreenup across all sites in both Europe and North America (Figure 2a), with a decreasing trend with rising latitude. This means there was more rapid canopy development in lower latitudes than in higher latitudes. However, Vgreenup demonstrated no clear patterns with longitude and altitude (Figure 2b,c). Furthermore, the above patterns were also confirmed when only considering sites in Europe or North America (Figure 2d–i). A previous study based on remote sensing observation found the latitude dependence of Vgreenup on both continents, but an opposite trend with latitude was observed in this study [21]. The discrepancies might be caused by the difference in the target scale between the satellite and field observations.

The spatial variation of Vgreenup was associated with the decoupling of the spatial patterns of its two components [19,22]. In this study, both Dgreenup and Agreenup were significantly correlated with Vgreenup along a spatial dimension, indicating that the spatial variations of Vgreenup were jointly regulated by Dgreenup and Agreenup (Figure 3a,b). In the whole study area, Vgreenup was more correlated with Agreenup (*R*^2^ = 0.83) than with Dgreenup (*R*^2^ = 0.61), which implies a more predominant effect of Agreenup variability compared with Dgreenup variability. This result is in line with the finding of a previous satellite-based study at a global scale [19]. Nevertheless, the variations of Dgreenup and Agreenup made different contributions to Vgreenup variation between continents. According to the correlation coefficients between Vgreenup and its components (Figure 3c–f), the spatial pattern of Vgreenup was more determined by Dgreenup in Europe but by Agreenup in North America.

This study further investigated the controls of the main spring climatic factors on the distributions of Vgreenup and its components (Dgreenup and Agreenup), by using the partial correlation. TA showed significant effects on Dgreenup (*R* = −0.51, *p* < 0.01), Agreenup (*R* = 0.71, *p* < 0.001) and Vgreenup (*R* = 0.66, *p* < 0.001) in the whole study area (Figure 4a). Specifically, increasing TA could shorten Dgreenup and promote Agreenup simultaneously, leading to an acceleration of Vgreenup. TA was reported to be the major driver for spring phenology based on long-term FLUXNET measurements [30]. Advanced SOG and the peak of the growing season (closing to EOG) in response to climate warming had been widely observed [10,31,32,33], but their non-uniform response magnitude would alter Agreenup. Under global warming, global average Agreenup decreased by 1.4 days during 1982–2015, according to the satellite LAI product [19]. Besides, TA had a stronger link with Agreenup (*R* = 0.71, *p* < 0.001) than with Dgreenup (*R* = −0.51, *p* < 0.01) in this study. The boosting effect of TA on Agreenup was consistent with the global greening and increasing productivity in recent decades due to climate warming [17,34,35]. Zhang et al., also reported the positive effect of increasing TA on the seasonal maximal gross primary productivity (GPP_max_) based on eddy covariance at an alpine meadow site [36]. Rising TA can promote plant growth by enhancing enzymatic activity when TA is lower than the optimum temperature [37]. With the positive effect of spring TA on Vgreenup, increasing spring TA could enhance spring GPP, which had been previously confirmed across 21 FLUXNET sites in temperate and boreal forests [38]. In this study, increasing SW also contributed to shorter Dgreenup and higher Vgreenup, similar to effects of TA (Figure 4a). SW is highly related to the heat requirement for leaf unfolding [39]. Lower solar radiation can significantly delay spring bud development by decreasing the accumulation of growing degree days [40]. Therefore, SW can influence Dgreenup and Vgreenup by altering the temperature sensitivity of SOG and EOG [41].

VPD and SM are commonly used indicators for atmosphere and soil dryness, respectively [42,43,44]. A significant effect by drought on vegetation greenup development was observed in this study. By increasing VPD or decreasing SM, Dgreenup and Agreenup tended to become longer and lower, respectively, resulting in the deceleration of Vgreenup for the whole study area (Figure 4a). Previous studies based on eddy covariance observations also demonstrated the reduction in photosynthesis caused by dryness [45,46]. GPP_max_ has also been reported to be enhanced by summer PRE, based on data from 24 AmeriFlux sites [47]. However, no significant relationship was found between spring PRE and Vgreenup in this study. This might be caused by the fact that vegetation growth was more directly affected by SM and VPD compared with PRE. Vegetation tends to lower photosynthesis by closing stomata in a high VPD condition [48,49]. Different from VPD, SM influences vegetation photosynthesis by controlling the absorption of soil nutrients. Increasing SM will facilitate plant nutrient absorption, leading to the stimulation of Vgreenup [50].

Besides, this study found that there were positive effects by wind speed on Agreenup and Vgreenup for the entire study area (Figure 4a). This might be explained by the fact that moderate wind speed would increase the gas exchange rate of leaf stomata, accelerating transpiration and intercellular CO_2_ exchange, which results in a higher intercellular CO_2_ concentration and improved photosynthesis capacity [51].

In this study, the climatic controls on the spatial variations of Vgreenup were also compared between Europe and North America. The temperature sensitivities of Agreenup and Vgreenup were greater in North America than in Europe (Figure 5). This difference in temperature sensitivity could be caused by the effects of climatic factors other than TA. The spatial variations of Vgreenup were jointly controlled by TA, SW, and VPD in Europe but only affected by TA in North America (Figure 5b,c). Without the limitations of solar radiation and water supply, the vegetation may have developed greater temperature sensitivities to maximize the thermal benefits [52,53,54].

ENF sites were included to investigate Vgreenup in this study. It is difficult to extract the phenological metrics from ENF by using NDVI or LAI, due to their narrow seasonal variations in vegetation greenness [55,56]. In comparison, GPP derived from eddy covariance has a great advantage in extracting the phenological metrics from ENF, which can help improve the estimation of Vgreenup for ENF. Besides, solar-induced chlorophyll fluorescence can reflect vegetation photosynthesis directly [57], which can be used to investigate the phenological metrics and Vgreenup for ENF in the future. VPD and SM are often coupled through land–atmosphere interactions, hindering the ability to predict ecosystem responses to dryness [58,59]. Several methods have been proposed to distinguish the effects of VPD and SM on vegetation growth [60,61,62]. Multi-methods may need to be synthesized for quantifying the relative contributions of VPD and SM to Vgreenup in future work.

## 4. Materials and Methods

### 4.1. Carbon Fluxes and Climatic Data

**Figure 6:**
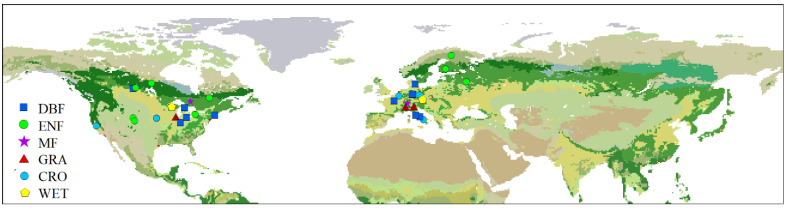
Spatial distribution of eddy covariance flux sites used in this study. DBF, deciduous broadleaf forest; ENF, evergreen needle-leaf forest; MF, mixed forest; GRA, grassland; CRO, cropland; WET, wetland.

Daily GPP data and climatic data were retrieved from the FLUXNET 2015 Tier 1 dataset (https://fluxnet.org/data/fluxnet2015-dataset/, accessed on 10 May 2022). The FLUXNET 2015 dataset provides in situ estimates of carbon, water, and heat fluxes between ecosystems and atmosphere of 212 sites across the globe, which are determined using the eddy covariance technique. The eddy covariance data for all the sites were processed by consistent and uniform procedures including quality control, gap-filling, and net ecosystem exchange (NEE) partitioning [63]. NEE measurements were partitioned into GPP and ecosystem respiration (ER). GPP based on nighttime partitioning method (GPP_NT_VUT_REF) was used in this study [64]. To avoid spurious effects caused by low-quality data, sites having at least 5 years with high-quality carbon flux data (NEE_VUT_REF_QC > 0.75 in a year) above 30° N were selected. In addition, only sites with mean start of greenup (SOG, defined below) occurring during spring (April–June) were included in this study. At last, 40 sites (417 site-year data) were selected to be analyzed, including 14 DBF sites, 10 ENF sites, 3 MF sites, 3 GRA sites, 8 CRO sites, and 2 WET sites (Figure 6, Table 1).

To analyze the relationship between Vgreenup and climatic variables, this study also collected the daily climatic data including TA, PRE, SW, WS, and VPD provided by the FLUXNET sites. Considering the unavailability of SM at most sites, the daily root-zone SM was extracted from Global Land Evaporation Amsterdam Model (GLEAM) v3.5a to reflect the soil water condition in root zone of each site (https://www.gleam.eu/, accessed on 15 May 2022) [65,66]. The depth of the root zone is different for low vegetation (0–100 cm) and tall vegetation (0–250 cm). The GLEAM v3.5a dataset was produced by combining satellite and reanalysis data, which provides temporally/spatially continuous SM at spatial resolution of 0.25° (~25 km), spanning from 1980 to 2020. The overall performance accuracy of GLEAM SM products is considerably high [43]. The mean values in spring were calculated for all climatic factors.

### 4.2. Calculation of Vgreenup

To remove outliers of GPP data, a Savitzky–Golay filter was adopted to smooth the daily GPP time series [67]. The width of the moving window was set to be 91 days, and the iteration time was set to be 1 according to a previous study [68]. Then, a modified double logistic function was used to fit the daily GPP for each year [32,69]:

(1) $$f\left(t\right)=\alpha_{1}+\frac{\alpha_{2}}{1+e^{-\partial_{1}\left(t-\beta_{1}\right)}}-\frac{\alpha_{3}}{1+e^{-\partial_{2}\left(t-\beta_{2}\right)}}$$

where *f*(*t*) is the daily GPP at day of year (DOY) *t*; $\alpha_{1}$ is the background GPP; $\alpha_{2}-\alpha_{1}$ and $\alpha_{3}-\alpha_{1}$ represent the amplitude relative to the background for the early summer plateau and the late summer plateau, respectively; $\partial_{1}$ and $\partial_{2}$ are the transition curvature parameters (normalized slope coefficients); and $\beta_{1}$ and $\beta_{2}$ are the midpoints in DOYs of these transitions for greenup and senescence, respectively.

**Figure 7:**
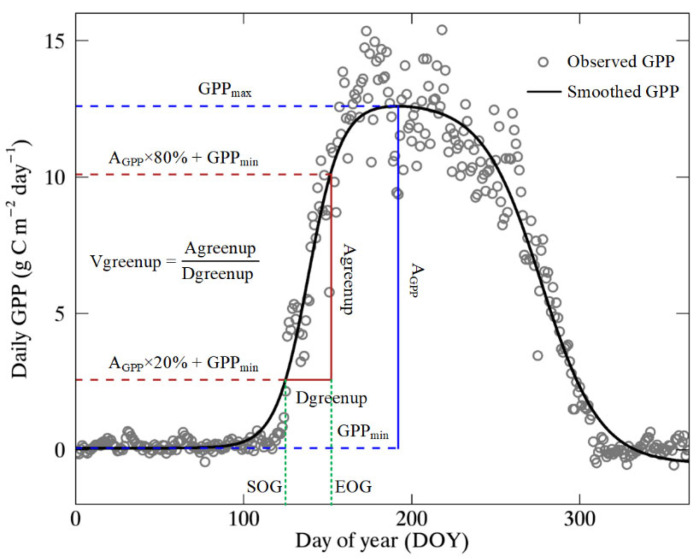
Scheme for determination of Dgreenup, Agreenup, and Vgreenup using daily GPP data at IT-Col site (deciduous broadleaf forest) in 2014. GPP_max_ and GPP_min_ represent the maximum and minimum smoothed GPP during the growth period, respectively. A_GPP_ represents the seasonal amplitude of GPP, which is the difference between GPP_max_ and GPP_min_.

The relative threshold method is a simple but practical method to extract phenological metrics [70]. In this study, the start and end of greenup period (SOG and EOG) were defined as the dates when the fitted GPP curve first reach 20% and 80% the seasonal amplitude, respectively [19]. Dgreenup was defined as the difference between EOG and SOG. Agreenup was calculated as the difference in GPP values at dates of EOG and SOG. At last, Vgreenup was calculated as the ratio of Agreenup to Dgreenup [19]. The process for determining Dgreenup, Agreenup, and Vgreenup is illustrated in Figure 7.

### 4.3. Statistical Analysis

Considering the limited time series of GPP, this study only analyzed the spatial variations rather than the temporal variations for Vgreenup. The indicators of Dgreenup, Agreenup, and Vgreenup were derived from each site-year. Then, the multi-year indicators were averaged at each site to analyze the spatial pattern of Dgreenup, Agreenup, and Vgreenup. One-way analysis of variance (ANOVA) and least significant difference (LSD) multiple comparisons test were performed to determine significant differences in Vgreenup among PFTs. The significant level was set at α = 0.05. Simple linear regression was adopted to analyze the relationship between Vgreenup and geographical factors (latitude, longitude, and altitude). Correlations between spatial anomalies of Vgreenup and its components (Dgreenup and Agreenup) were analyzed to identify whether the spatial variations in Vgreenup were affected by Dgreenup and Agreenup. Partial correlation analysis was also conducted to evaluate the isolated effects of spring climatic factors (TA, PRE, SW, VPD, WS, and SM) on Dgreenup, Agreenup, and Vgreenup. The statistical significances of the regression and correlation coefficients were examined using the t-test. The *p*-values less than 0.05 were considered significant. The direction and magnitude regarding driving factors on spatial variations of Vgreenup were compared between Europe and North America. Besides, the results in this study were also compared with the relevant findings in previous studies.

## 5. Conclusions

Based on 417 site-years’ flux data from 40 sites in the northern temperate and boreal regions, this study examined the characters of Vgreenup for different PFTs and further investigated the roles of climatic variables in controlling the spatial variation of Vgreenup. The results found that CRO and ENF had the largest and lowest Vgreenup among all PTFs, respectively. Vgreenup showed strong dependence on latitude but no obvious dependence on longitude or altitude. The spatial variations of Dgreenup and Agreenup jointly controlled the distribution of Vgreenup but made distinct contributions to Vgreenup variation between Europe and North America. Different controls of spring climatic factors on the spatial variation of Vgreenup were also observed between Europe and North America. In Europe, TA and SW had a positive effect on Vgreenup, while VPD showed a negative influence. However, Vgreenup was only affected by TA in North America. Such a difference led to a higher temperature sensitivity for Vgreenup in North America than in Europe. This study can help us to better understand the development of vegetation greenup from the perspective of field observation, which is an important supplement and validation for satellite-based research. Besides, long-term field observation is needed to further investigate the temporal dynamics of Vgreenup, for a more comprehensive analysis in the future.

## Figures and Tables

**Table 1:** Statistics of Vgreenup for each site used in this study.

Site	IGBP	Latitude (°)	Longitude (°)	Altitude (m a.s.l.)	Vgreenup (g C m^−2^ day^−2^)	Observation Period	Continent
BE-Lon	CRO	50.55	4.75	167	0.30 ± 0.05	2005–2014	EU
CA-Gro	MF	48.22	−82.16	340	0.12 ± 0.03	2004–2013	NA
CA-Man	ENF	55.88	−98.48	259	0.09 ± 0.03	1995–1996, 1998, 2000–2003	NA
CA-Oas	DBF	53.63	−106.20	530	0.26 ± 0.03	1997–2010	NA
CA-Obs	ENF	53.99	−105.12	629	0.08 ± 0.01	2000–2010	NA
CA-Qfo	ENF	49.69	−74.34	382	0.06 ± 0.01	2004–2010	NA
CA-TP3	ENF	42.71	−80.35	184	0.13 ± 0.02	2008–2014	NA
CA-TP4	ENF	42.71	−80.36	184	0.13 ± 0.04	2003–2014	NA
CH-Lae	MF	47.48	8.36	689	0.15 ± 0.03	2005–2014	EU
CZ-wet	WET	49.02	14.77	426	0.19 ± 0.05	2007–2014	EU
DE-Geb	CRO	51.10	10.91	162	0.22 ± 0.08	2001–2014	EU
DE-Hai	DBF	51.08	10.45	430	0.23 ± 0.04	2000–2012	EU
DE-Kli	CRO	50.89	13.52	478	0.23 ± 0.04	2005–2007, 2010–2012, 2014	EU
DE-Lnf	DBF	51.33	10.37	451	0.25 ± 0.03	2003–2006, 2010–2012	EU
DK-Sor	DBF	55.49	11.64	40	0.28 ± 0.03	1997–2013	EU
FI-Hyy	ENF	61.85	24.29	181	0.11 ± 0.02	1997–2014	EU
FI-Sod	ENF	67.36	26.64	180	0.09 ± 0.02	2001, 2003–2014	EU
FR-Fon	DBF	48.48	2.78	103	0.23 ± 0.03	2005–2013	EU
IT-BCi	CRO	40.52	14.96	20	0.53 ± 0.07	2005–2009	EU
IT-Col	DBF	41.85	13.59	1560	0.25 ± 0.04	1998, 2001, 2007–2009, 2011, 2014	EU
IT-Mbo	GRA	46.01	11.05	1550	0.28 ± 0.04	2003–2013	EU
IT-Ro1	DBF	42.41	11.93	235	0.19 ± 0.03	2001–2006, 2008	EU
IT-Ro2	DBF	42.39	11.92	160	0.26 ± 0.03	2002, 2004–2007, 2010, 2012	EU
IT-Tor	GRA	45.84	7.58	2160	0.17 ± 0.04	2009–2014	EU
RU-Fyo	ENF	56.46	32.92	265	0.14 ± 0.03	1999–2009, 2012–2014	EU
US-GLE	ENF	41.37	−106.24	3197	0.10 ± 0.04	2006–2014	NA
US-Ha1	DBF	42.54	−72.17	340	0.21 ± 0.04	1992–2001, 2003–2004, 2006–2007, 2009–2012	NA
US-IB2	GRA	41.84	−88.24	227	0.22 ± 0.04	2005–2011	NA
US-Los	WET	46.08	−89.98	480	0.17 ± 0.03	2001–2006, 2014	NA
US-MMS	DBF	39.32	−86.41	275	0.25 ± 0.02	1999–2014	NA
US-NR1	ENF	40.03	−105.55	3050	0.11 ± 0.01	2000–2014	NA
US-Ne1	CRO	41.17	−96.48	361	0.54 ± 0.03	2002–2012	NA
US-Ne2	CRO	41.16	−96.47	362	0.47 ± 0.15	2003–2012	NA
US-Ne3	CRO	41.18	−96.44	363	0.38 ± 0.13	2002–2012	NA
US-Oho	DBF	41.55	−83.84	230	0.29 ± 0.02	2004–2006, 2008–2013	NA
US-Pfa	MF	45.95	−90.27	470	0.14 ± 0.03	1997–2004, 2006–2009, 2011–2014	NA
US-Twt	CRO	38.11	−121.65	-7	0.26 ± 0.06	2000–2014	NA
US-UMB	DBF	45.56	−84.71	234	0.22 ± 0.04	2000–2014	NA
US-Umd	DBF	45.56	−84.70	239	0.23 ± 0.02	2008–2014	NA
US-WCr	DBF	45.81	−90.08	520	0.24 ± 0.05	2000–2003, 2005–2006, 2011–2014	NA

DBF, deciduous broadleaf forest; ENF, evergreen needle-leaf forest; MF, mixed forest; GRA, grassland; CRO, cropland; WET, wetland; EU, Europe; NA, North America.

## Data Availability

Publicly available datasets were analyzed in this study. The data source and access links are indicated in the text.
